# Determinants of severe acute malnutrition among under 5 children in Satar community of Jhapa, Nepal

**DOI:** 10.1371/journal.pone.0245151

**Published:** 2021-02-03

**Authors:** Kajol Dahal, Deepak Kumar Yadav, Dharanidhar Baral, Birendra Kumar Yadav

**Affiliations:** 1 School of Public Health and Community Medicine, B.P Koirala Institute of Health Sciences, Dharan, Nepal; 2 Faculty of School of Public Health and Community Medicine, B.P Koirala Institute of Health Sciences, Dharan, Nepal; International Centre for Integrated Mountain Development (ICIMOD), Kathmandu, Nepal, NEPAL

## Abstract

**Background:**

Severe acute malnutrition (SAM) is the most extreme and visible form of undernutrition plagued by chronic poverty, household food insecurity, lack of education. One of the indigenous and marginalized community of Nepal, Satar/Santhal has often been neglected and is devoid of good education and are economically deprived. This predisposes under 5 children of Satar into malnutrition. The study aims to assess determinants of SAM among children under 5 years of age in Satar community of Jhapa district, Nepal.

**Material & methods:**

A community based matched case control study was carried from September 2019 to February 2020 among under five children of Satar community residing in Jhapa district. Multistage random sampling technique was used to select 50 cases and 100 controls in the ratio of 1:2. Information was collected through personal interview with the parents and anthropometric measurement of the children was measured. Bivariate and multivariate conditional logistic regression analysis was used to explore the determinants of severe acute malnutrition.

**Results:**

A total of 664 children between the age group of 6–59 months were screened for SAM. The prevalence of SAM was found 7.53%. Factors like, low economic status, birth interval less than 2 years, frequency of breast feeding <8 times/day and household food insecurity were found to be significant determinants of SAM. Multivariate logistic regression documented low economic status (AOR: 11.14, 95% CI 1.42 to 87.46); and frequency of breast feeding <8 times/day (AOR: 2.09, 95% CI 1.00 to 4.37) as determinants of SAM.

**Conclusion:**

Low economic status and frequency of breast feeding less than 8times/day were major determinants of SAM among children under 5yrs of age. Ending malnutrition will require greater efforts and integrated approaches to eradicate extreme poverty. Multi-sector approaches have been conducting for SAM in Nepal but there are no specific approaches for marginalized community.

## Background

The world is marching towards the elimination of infectious disease and hunger but developing world is still fighting with malnutrition and its associated morbidities. The term malnutrition is multifaceted which encompasses both over nutrition and under nutrition [1]. Severe acute malnutrition is the most extreme and visible form of undernutrition, caused not from emergencies but plagued by chronic poverty, household food insecurity, lack of education [1,2]. It is defined by very low weight for height (below -3 z scores of the median WHO growth standards), by visible severe wasting, or by the presence of nutritional oedema [3].Severely malnourished children have been estimated to have a greater than nine fold increased risk (relative risk of 9.4) of dying compared to a well-nourished child [4].

Globally, 52 million children under 5 years of age are wasted and of those 17 million are severely wasted; around 45% of deaths among children under 5 years of age are linked to under nutrition, which mostly occur in low and middle-income countries [5]. Asia is home to the largest number of children under 5 years of age with SAM, which is a major hindrance to optimal human capital development [6]. The consequences of malnutrition are serious and life-long, falling hardest on the very poor and on women and children [7]. Under nutrition interacts with repeated bouts of infectious disease; causing an estimated 3.5 million preventable maternal and child deaths annually, and its economic costs in terms of lost national productivity and economic growth are huge [4,8].

Based on WHO estimates, all forms of malnutrition accounts for more than 50 per cent of child mortality in Nepal and wasting prevalence is at a critical level in Nepal, affecting an estimated 430,000 children under five years of age at any point in time [8]. As per Nepal Demographic and Health Survey (NDHS) 2016, 2 per cent under-five year old children in Nepal are suffering from SAM (severely wasted: too thin for their height) [9]. Similarly, a study conducted in Bara district of Nepal showed the prevalence of SAM in under 5 children to be 4.14% and another study done in Nepal found 5.8% prevalence [10,11].

Several studies have found the association between SAM and low socio economic status [12], late initiation of breast feeding after one hour of the birth, non-exclusive breast feeding, birth interval less than two years [13], mother’s age at birth [10], maternal education [14], and family size [11]. Along with malnutrition among children and women, food insecurity remains an alarming problem in Nepal [15]. According to Nepal Health Demographic Survey 2016, 52% of the households were food insecure. In the 2019 Global Hunger Index, Nepal ranks 73rd out of 117 qualifying countries and with a score of 20.8, Nepal suffers from a level of hunger that is serious [16]. Similarly, a study conducted in two Terai districts of Nepal (Jhapa and Bara) found that children from severely food insecure households were four times more likely to be severely malnourished [11].

There is a problem of malnutrition in general population, but the problem in indigenous people is worse. The Santhal (Satar) community, one of the most excluded ethnic groups in Nepal, is concentrated in Jhapa and Morang districts in Eastern Nepal [17]. There are studies done in West Bengal, India, which has found health and nutritional standards of children in Santhal community, remain to be unsatisfactory and girls were more prone to have undernutriton than boys [18–20]. Poverty, inequalities are still the pillars that hinder them from progressing. Because of such socioeconomic inequalities in these indigenous communities and poverty being a major risk factor of severe acute malnutrition, Satar community was chosen as study population. And to our best knowledge, this study is the first of its kind in Nepal to investigate the predictors of severe acute malnutrition in such a marginalized and vulnerable population in Nepal. Therefore, the study aims to assess the determinants of severe acute malnutrition (SAM) among children under 5 years of age in Satar community of Jhapa district, Nepal.

## Methods

The study type of this research is: Human Subject Research (involving human participants) Institutional Review Committee (IRC) of B.P Koirala Institute of health Sciences, Dharan, Nepal approved the study. The approval number of this research is: 015/076/077-IRC. The form of consent was obtained (written) from the participants.

### Study area

This study was conducted among Satar children aged 6–59 months from Jhapa district of Nepal. Jhapa is one the eastern district of Nepal located in Province 1. Satar (Santhal) is an indigenous community, concentrated in Jhapa and Morang districts of Eastern Nepal [21].

### Study design, duration and selection criteria

A community based matched case-control study was conducted from September 2019 to February 2020. Age and sex matching was done among the cases and controls. The cases were the severely acute malnourished children below the age of 5years ascertained by using the weight for height Z-score< −3SD below the median according to WHO 2006 growth standard and the controls were sex and age-matched children aged 6–59 months from same community without malnutrition [22]. Children below five years from Satar (Santhal) community residing in Jhapa district of Nepal whose guardian gave consent were included in our study. Those children who were severely ill or who were with co-morbidities were excluded from the study.

### Sample size and sampling technique

The sample size was calculated using the formula given by Schlesselman 1982 [23] assuming 5% level of significance, 90% power (1-β) and case control ratio of 1:2. Based on the case control study done in Bara, Nepal the percentage of controls exposed for bottle-feeding was assumed at 18% with an odds ratio of 4.56 [10]. Assuming 10% non-response rate, the sample size was calculated to be 150; 50 cases and 100 controls.

A multi-stage random sampling technique was used to select under 5 children. First, Jhapa district was purposively selected. It consists of 8 urban and 7 rural municipalities. Among these fifteen municipalities of Jhapa district two urban (Birtamode and Bhadrapur) and two rural (Jhapa and Haldibari) were selected using simple random technique (lottery method) to give a total of four municipalities. In these municipalities, 664 children aged 6–59 months were identified whose anthropometric measurements (weight and height) were taken and compared with WHO 2006 simplified growth standards and were categorized as cases (weight for height < −3SD) or controls (weight for height > −2SD).

The cases and controls were matched in a 1:2 ratio with similar age intervals of (6–8, 9–12, 12–14, 15–17, 18–20, 21–23, 24–26, 27–29, 30–32, 33–35, 36–38, 39–41, 42–44, 45–47, 48–50, 51–53, 54–56, and 57–59) months and sampling frames of cases or controls were prepared for each municipality. Using simple random technique (lottery method), the required number of cases and controls were then selected. Proportional allocation method was employed for adequate representation of cases and controls from each municipality. Only one younger child was selected per household during data collection.

### Questionnaire

A semi-structured questionnaire was developed based on study objectives. A validated Nepal Demographic and Health Survey 2016 (NDHS) questionnaire was adapted for categorizing socio-economic status, educational level and feeding practices [9]. Household Food Insecurity Access Scale (HFIAS) measurement tool was used to collect information on food insecurity at household level developed by the Food and Nutrition Technical Assistance Project (FANTA) [24]. Pilot testing was done in similar setting in Biratnagar, Nepal. The questionnaire was pretested on 10% of the sample size; 15 children (5 with SAM and 10 without SAM), and necessary corrections were made.

### Variables

SAM was identified using the weight for height Z-score< −3SD below the median for children aged 6–59 months according to WHO 2006 growth standard [22]. Independent variables were categorized into socio-demographic variables, feeding practices and household food insecurity. A robust literature review was done to group potential factors associated with SAM. Socio-demographic factors included age and sex of child, religion, mother and father’s educational level, mother’s age at birth of the child, family Income, number of family members and birth Interval. Majority of the mothers of under 5 children in indigenous community of Satar were unable to show child health card or birth certificate for verifying date of birth. Therefore, the date of birth of the child was asked and its validity was verified through sibling method (birth order of child), local feast and festival calendar. The reliability was checked with other supportive questions like age of mother at pregnancy, and age of mother at present. Family income <1.90$/day were taken to be below poverty line [25]. Feeding practices included colostrum feeding, initiation of breast feeding, frequency of breast feeding, exclusive breast feeding upto 6 months, bottle feeding, initiation of complementary feeding. Initiation of breast-feeding was categorized as within 1 hour or after 1 hour of birth. Initiation of complementary feeding refers to introduction of additional foods other than the breast milk at 6 months, which was categorized into before 6 months or at 6 months or after 6 months. Household food insecurity was classified into severely/moderately/mildly food insecure or food secure.

### Data collection

Face to face interview was conducted with mother of eligible children using paper based semi-structured questionnaire. Any inconsistencies in questionnaire were addressed during the fieldwork. Anthropometric measurements (weight and recumbent length/height) of the children were taken and compared with height for weight indicators Z-score as per WHO 2006 growth standards. For weight, a digital scale was used for accurate measurement with minimal clothing. The height measurement was taken on flooring that was not carpeted and against a flat surface such as a wall. The measurements were done for 3 times to finalize final measurement to record. Validation of instruments and measurements was done on a daily basis.

### Statistical analysis

The collected data were entered into Microsoft Excel, 2007 and were converted into Statistical Package for Social Sciences (SPSS 11.5 version) for data analysis.

Descriptive data were presented as frequency, percentage, mean and standard deviation. The association between independent and outcome variable was analyzed using bivariate and multivariate conditional logistic regression analysis. Statistical significance was tested with 95% confidence interval and a value of p<0.05 was considered significant. Calculation of adjusted odds ratio (AOR) was done in order to measure the net effect size of the entered variables. Goodness of fit was tested using Hosmer and Lemeshow test. The conditional logistic regression model is given by logit(p) = α_(stratum)i_+β_1_x_1_+β_2_x_2_+β_k_x_k_ where p is probability of being a case, β_1_X_1_ to β_k_X_k_ are the regression coefficients that represent log odds and α_1_ to α_i_ denotes contribution to the logit of all terms constant within the ith matching set; the interval matching variables need to be controlled in the conditional model because the matching process makes cases and controls similar not only for the matching variables but also for the exposure status [26,27]. A multi-collinearity diagnostic test was applied between the independent variables before logistic regression was applied. Decisive criteria was set out at variance inflation factor (VIF) value of >3. The variables within the criteria were only used for logistic regression.

### Ethical clearance

It was obtained from the Institutional Review Committee (IRC) of B.P Koirala Institute of Health Sciences (BPKIHS), Dharan Nepal. Written consent was obtained from the municipalities of Jhapa district. The purpose of the study and the procedure was well explained and written informed consent was gained for publication as well before starting the data collection. The participants were also informed that their participation was voluntary, and can withdraw at any moment. Moreover, they were assured regarding their anonymity, and the confidential treatment of their responses. Those children identified as SAM were given proper nutritional counseling and were also advised to seek treatment for the condition.

## Results

The screening was carried out in 664 children, aged 6–59 months to find out 50 cases of SAM in the indigenous community of Jhapa district. The prevalence of SAM among under 5 children was found to be 7.53%. Table 1 provides the description of socio-demographic characteristics among cases and controls. The study was age and sex matched. Most (92% of cases and 89% of controls) of the study participants belonged to Hindu religion. The study showed that majority (37.3% of mother and 28.7% of father) of under 5 children parents were illiterate. The mean±SD ages of the mothers at the time of delivery of the cases and controls were 22.20±3.49 and 21.87±3.53 years respectively. And, the mean±SD of the number of family members of the cases and controls were 5.02±1.74 and 4.64±1.40 respectively. In total there were 84% of families below poverty line (earning <$1.9/day). Likewise, majority (20%) of the cases were born in less than 2 years of birth interval compared to controls (8%). Almost all, 92% of the cases and 93% of the controls were colostrum fed. Nearly two-third (73.3%) of the children were initiated with breast feeding within 1 hour of birth but almost all of the children (95.5%) were not exclusively breastfed. More than half of the participants (54%) of the cases were breastfed less than 8 times a day, compared with controls (30%). Only 26% of the cases were bottle fed in comparison to controls (31%). The overall prevalence of household food insecurity access was 72%. Majority 84% of the cases and two-third (66%) of the controls were severely food insecure. Only 10% of the cases and nearly one-third of the controls were secured for food.

**Table 1:** Description of socio-demographic characteristics of cases and controls.

Characteristics	Cases			Controls				Total	
	N = 50	%		N = 100			%	N = 150	%
**Age of the child**									
6–17 months	20	40.0		40		40.0		60	40.0
18–29 months	15	30.0		30		30.0		45	30.0
30–41 months	6	12.0		12		12.0		18	12.0
42–53 months	3	6.0		6		6.0		9	6.0
54–59 months	6	12.0		12		12.0		18	12.0
**Sex of the Children**									
Male	23	46.0		46	46.0			69	46.0
Female	27	54.0		54	54.0			81	54.0
**Religion of the families**									
Hindu	46	92.0		89	89.0			135	90.0
Christian	4	8.0		11	11.0			15	10.0
**Mother's Education Level**									
Illiterate	24	48.0		32		32.0		56	37.3
Informal Education	7	14.0		14		14.0		21	14.0
Primary Education	11	22.0		26		26.0		37	24.7
Secondary Education	5	10.0		20		20.0		25	16.7
SLC and Above	3	6.0		8		8.0		11	7.3
**Father's Education Level**									
Illiterate	17		34.0	26	26.0			43	28.7
Informal Education	11		22.0	13	13.0			24	16.0
Primary Education	8		16.0	23	23.0			31	20.7
Secondary Education	9		18.0	20	20.0			29	19.3
SLC and Above	5		10.0	18	18.0			23	15.3
**Mother's age at delivery (yrs)**	22.20		±3.49	21.87	±3.53			21.98	±3.5
**No. of family members**	5.02		±1.74	4.64	±1.40			4.77	±1.5
**Economic Status**									
Below Poverty Line	49	98.0		77		77.0		126	84.0
Above Poverty Line	1	2.0		23		23.0		24	16.0
**Birth Interval**									
First Birth	16	32.0		48		48.0		64	42.7
Less than 2 years	10	20.0		8		8.0		18	12.0
More than 2 years	24	48.0		44		44.0		68	45.3
**Colostrum Feeding**									
Yes	46	92.0		93	93			139	92.7
No	4	8.0		7	7.0			11	7.3
**Initiation of Breast Feeding**									
Within 1 hour of birth	40	80.0		70	70.0			110	73.3
After 1 hour of birth	10	20.0		30	30.0			40	26.7
**Exclusive Breast Feeding**									
Done	1	2.0		6		6.0		7	4.7
Not Done	49	98.0		94		94.0		143	95.3
**Initiation of Complementary Feeding**									
Before 6 months	10	20.0		15		15.0		25	16.7
At 6 months	11	22.0		33		33.0		44	29.3
After 6 months	29	58.0		52		52.0		81	54.0
**Frequency of Breast Feeding**									
<8/day	27	54.0		30		30.0		57	38.0
≥8/day	23	46.0		70		70.0		93	62.0
**Bottle Feeding**									
Yes	13	26.0		31		31.0		44	29.3
No	37	74.0		69		69.0		106	70.7
**Household Food Insecurity Access Category**									
Severely Food Insecure	42	84.0		66		66.0		108	72.0
Moderately Food Insecure	2	4.0		0		0.0		2	1.3
Mildly Food Insecure	1	2.0		2		2.0		3	2.0
Food Secure	5	10.0		32		32.0		37	24.7

The bivariate analysis showed that economic status, birth interval, frequency of breast feeding, and household food insecurity were significantly associated with SAM (Table 2).

**Table 2:** Association between severe acute malnutrition and variables among under five children in Satar community of Jhapa district (n = 150).

Characteristics	Cases		Controls		COR (95% CI)*	p Value*
	n = 50/ mean	% / SD	n = 100/mean	% / SD		
**No. of family members**	5.02	±1.74	4.64	±1.40	-	0.168
**Mother's Educational Level**						
Illiterate	24	48.0	32	32.0	2.534(0.978–6.566)	0.056
≤Primary Education	18	36.0	40	40.0	1.560(0.609–4.002)	0.354
≥Secondary Education	8	16.0	28	28.0	Ref	
**Economic Status**						
Below Poverty Line	49	98.0	77	77.0	13.103(1.732–99.149)	**0.013**
Above Poverty Line	1	2.0	23	23.0	Ref	
**Birth Interval**						
Less than 2 years	10	20.0	8	8.0	2.677(1.011–7.089)	**0.048**
First birth or >2 years	40	80.0	92	92.0	Ref	
**Initiation of Complementary Feeding**						
<6 or >6 months	39	78.0	67	67.0	1.782(0.808–3.932)	0.153
At 6 months	11	22.0	33	33.0	Ref	
**Frequency of Breast Feeding (times)**						
<8/day	27	54.0	30	30.0	2.612(1.282–5.321)	**0.008**
≥8/day	23	46.0	70	70.0	Ref	
**Household Food Insecurity Access**						
Food Insecure	45	90.0	68	68.0	4.272(1.454–12.549)	**0.008**
Food Secure	5	10.0	32	32.0	Ref	

However, after the multivariate analysis, economic status and frequency of breast-feeding were found to be strong predictors of SAM (Table 3). The odd of having SAM was 11.14 folds more among the under 5 children of Satar/Santhal families who were below the poverty line than those who were above the poverty line (95% CI: 1.42–87.46). Similarly, children who were breastfed <8times/day (AOR: 2.04, 95% CI: 1.00–4.37) were more likely to suffer from SAM than those who were breastfed more than 8 times perday.

**Table 3:** Multivariate conditional logistic regression analysis to find out the determinants of severe acute malnutrition among the under five children in Satar community of Jhapa district (n = 150).

Characteristics	β coefficient	COR(95% CI)	AOR(95% CI)	p Value
**Economic Status**				
Below Poverty Line	2.410	13.103(1.732–99.149)	11.139(1.419–87.456)	**0.022**
Above Poverty Line	Ref			
**Frequency of Breast Feeding**				
<8/day	0.738	2.612(1.282–5.321)	2.093(1.003–4.368)	**0.049**
≥8/day	Ref			

## Discussion

The study aimed to assess the determinants of severe acute malnutrition (SAM) among children under 5 years of age in Satar community of Jhapa district, Nepal. A total of 664 children were approached and screened. The overall prevalence of severe acute malnutrition (SAM) was found to be 7.53%. The finding was consistent with the study conducted in Sudan and WHO prevalence of severe wasting in South East Asia in 2017 [7,28]. However, study conducted in India and recent data of NDHS, Nepal showed a comparatively low prevalence of SAM [9,29]. The higher prevalence seen might be due to the study population, Satar community, which is vulnerable and marginalized community of Nepal wherein the Santhal children are still devoid of good feeding practices, and economic stability.

In this study, mother’s age at birth was not an independent determinant for SAM, which is consistent with studies conducted in Rupandehi, Nepal and Ethiopia [30,31]. The possible reason might be that early marriage was very common in the Satar community; women were prepared for all the difficulties generally experienced by new mothers (especially young mothers) in taking care of a household, child bearing and rearing. Similarly, mother’s educational level was not significantly associated with SAM. The result was in divergence with the study conducted in Illam, Nepal, Pakistan and Bangladesh [32–34]. Adherence of mother to the cultural practices prevailing in the Satar community might have influenced the feeding practices rather than the educational level of the mother. Likewise, father’s educational level was also not an independent determinant for SAM and the studies done in India and Kenya supports our finding [35,36]. The possible explanation might be that fathers of Satar children were often busy with the external chores, which limited their involvement in any decision or providing care towards their children.

The study revealed that low economic status (below poverty line; earning <$1.9/day)) was more than 11 folds at risk (AOR: 11.139 at 95% CI: 1.419–87.456) of developing SAM in children less than 5 years, which was statistically significant. The finding of our study is supported by the study done in Odisha of India, Bangladesh, and Ghana [29,34,37]. This could be explained by the fact that children from families of low socioeconomic status have limited access to food, health services, hygiene and sanitation. However, studies conducted in Illam, Nepal and Kenya showed that low economic status was not statistically significant with malnutrition [32–36].

In our study there was no significant association between SAM and number of family members. The study done in Rupandehi, Nepal, Bangladesh, Pakistan are in accordance with the result of our study [30,38,39]. The insignificant difference found between SAM and family size might be due to the social setting of the indigenous village (Satar), where children of both cases and controls live in joint family.

Birth interval was found significantly associated with SAM in bivariate analysis of our study but upon adjusting confounders in multivariate analysis, it was not significantly associated with SAM. In accordance with our study, a study done in Africa has also found birth interval to be insignificantly associated with SAM [40]. Other driving factors like poor economic condition of the household might have impacted more on the occurrence of SAM, therefore, birth interval was found to be insignificant with SAM.

The study showed that colostrum feeding, initiation of breastfeeding, exclusive breastfeeding, initiation of complementary feeding and bottle-feeding were not statistically significant with SAM and the results are in line with study conducted in Ethiopia and Nepal [30,31,41] but not studies from India, Nigeria, and Pakistan [12,42,43]. There was not much variation seen between the feeding practices of cases and controls, therefore, the association was not observed.

However, evidence from our study indicated that, children who were breast fed less than 8times/day were more than two folds at risk of developing SAM (AOR: 2.093, at 95% CI: 1.003–4.368) which is in convergent to another case control study conducted in Nepal [10] and a study done in India [12]. In contrary to our finding, a study from Ethiopia found no association between frequency of breast feeding and SAM [13]. The possible explanation for low frequency of breast-feeding might be that most of the Satar’s families are below the poverty line, which compels mother to work for daily earning. Therefore, the time spent at home of the mother affects breastfeeding frequency and babies do not get breast-fed whenever they desire for. Also, the lack of adequate nutrition in mothers limits sufficient milk production to feed their children as per requirement. Finally, food insecurity was not an independent determinant for SAM, which is in line with the studies conducted in Ethiopia and Uganda [44,45].

## Conclusion

Economic status and frequency of breast-feeding were found to be strong predictors of SAM, therefore, multi-faceted and multi-pronged strategies should be employed from government, stakeholders and political leaders to address the nutritional needs of deprived population. Policies that promotes and provides income-generating opportunities to the marginalized communities should be strongly emphasized. Furthermore, community based intervention of new-born care must be strengthened through the mobilization of female community health volunteers, community health workers etc. who will underpin the importance of breast-feeding practices in the deprived communities.

## Supporting information

S1 File (DOCX)

S2 File (DOCX)

S3 File (DOCX)

## Abbreviations

AOR: Adjusted Odds Ratio
BPKIHS: B.P. Koirala Institute of Health Sciences
CI: Confidence Interval
COR: Crude odds ratios
NDHS: Nepal Demographic and Health Survey
SAM: Severe Acute Malnutrition
SD: Standard Deviation
SLC: School Leaving Certificate
UNICEF: United Nations International Children's Emergency Fund
WHO: World Health Organization
