# Supplementary material for: Determinants of severe acute malnutrition among under 5 children in Satar community of Jhapa, Nepal
Source: PLoS One. 2021 Feb 3;16(2):e0245151. doi: 10.1371/journal.pone.0245151 (PMC7857586; doi:10.1371/journal.pone.0245151)
Supplement: S1 File — (DOCX) [file pone.0245151.s001.docx]

**jL=kL= sf]O/fnf :jf:Yo lj1fg k|lti7fg**

**w/fg, g]kfn**

## ;j]{If0f ljj/0f

**ldltM**

**s_ hg;f+lVosL, ;fdflhs / cfly{s sf/sx?M**

| **sf]8** | **k\|Zgx?** | **k\|ltls\|of** |
| --- | --- | --- |
| **!_** | **aRrfsf] pd]/ -dlxgfdf_** |  |
| **@_** | **lnË** | □ k'?if □ dlxnf □ cGo |
| **#_** | **wd{** | □ lxGb' □ ls\|lZrog □ d'l:nd  □ cGo |
| **$_** | **cfdfsf] z}lIfs :t/** | □cgk9 □cgf}krfl/s lzIff □ k\|fylds □dfWolds □ P;Pn;L / dfly |
| **%_** | **a'afsf] z}lIfs :t/** | □cgk9 □cgf}krfl/s lzIff □ k\|fylds □dfWolds □ P;Pn;L / dfly |
| **^_** | **aRrf hGdfpFbf cfdfsf] pd]/** |  |
| **&_** | **kl/jf/sf] cfo** | ==================-dfl;s_  ==================-jflif{s_ |
| ***_** | **Kl/jf/sf] ;b:osf] ;+Vof** |  |
| **(_** | **hGd cGt/fn** | □ klxnf] hGd □ b'O{ jif{eGbf a9L  □ b'O{ jif{eGbf yf]/} |

**aRrfsf] vfglkgsf] cEof;x? M**

| **sf]8** | **k\|Zgx?** | **k\|ltls\|of** |
| --- | --- | --- |
| **!_** | **cfdfsf] klxnf] kx]nf] -lauf}tL_ b'w v'jfPsf]<** | □ xf] □ xf]Og |
| **@_** | **:tgkfgsf] ;'?jft** | □ hGd]sf] Ps 306fleq  □ hGd]sf] Ps 306fkl5 |
| **#_** | **k"0f{ :tg kfg ^ dlxgf;Dd** | □;DkGg u/]sf] □ ;DkGg gu/]sf] |
| **$_** | **k'/s kfgsf] ;'?jft -cfGg k\|f;g_** | □ ^ dlxgf eGbf cl3 □^ dlxgfdf  □ ^ dlxgf kl5 □ cGo |
| **%_** | **lbgdf:tgkfgsf] cfj[lt** | □lbgdf * k6s eGbf sd  □lbgdf * k6s jf ;f] eGbf a9L |
| **^_** | **af]6n kfg** | □ u/]sf] xf]] □ u/]sf] xf]Og |

**aRrfsf] zf/Ll/s dfkg**

| **aRrf prfO** | =======-l;Pd_ |
| --- | --- |
| **aRrf tf}n** | ========-s]hL_ |

**kfl/jfl/s vfB c;'/Iftf kx'Fr :t/**

| g+= | k\|Zgx? | k\|ltls\|ofsf ljsNkx? |
| --- | --- | --- |
| != | Uft rf/ xKtfdf, s] xh'/nfO{ vfBfGgsf] ck'u xf]nf eGg] lrGtf nfUof] < | != nfu]g -@ gDa/sf] pQ/ lbg' xf]nf _ @= nfUof] |
| !=s | of] k\|fo slt eO/xG5 < | != Pdbd sd -ut rf/ xKtfdf Ps jf b'Ok6s_  @ = slxn]sfxLF -ut rf/ xKtfdf tLg b]lv bz k6d;Dd_  # = k\|fo -ut rf/ xKtfdf bz k6s eGbf w]/}_ |
| @. | Uft rf/ xKtfdf, s] xh'/ jf xh'/sf] kl/jf/sf] ;b:onfO{ >f]tsf] sdLn] ubf{ s'g} k\|sf/sf] vfBfGgsf] c;'ljwf eof] < | != ePg - # gDa/sf] pQ/ lbg' xf]nf_ @=eof] |
| @=s | of] k\|fo slt eO/xG5 < | ! = Pdbd sd -ut rf/ xKtfdf Ps jf b'Ok6s_  @ = slxn]sfxLF -ut rf/ xKtfdf tLg b]lv bz k6d;Dd_  # = k\|fo -ut rf/ xKtfdf bz k6s eGbf w]/}_ |
| #= | Uft rf/ xKtfdf, s] xh'/ jf xh'/sf] kl/jf/sf] ;b:onfO{ >f]tsf] sdLn] l;ldt dfqfdf dfq vfBfGgsf] ;'ljwf eof] < | != ePg -$ gDa/sf] pQ/ lbg' xf]nf_ @= eof] |
| #=s | of] k\|fo slt eO/xG5 < | ! = Pdbd sd -ut rf/ xKtfdf Ps jf b'Ok6s_  @ = slxn]sfxLF -ut rf/ xKtfdf tLg b]lv bz k6d;Dd_  # = k\|fo -ut rf/ xKtfdf bz k6s eGbf w]/}_ |
| $= | Uft rf/ xKtfdf, s] xh'/ jf xh'/sf] kl/jf/sf] ;b:onfO{ >f]tsf] sdLn] cfkm"nfO{ dg nfu]sf], dg gkg{] vfBfGg vfg'kof{] < | != k/]g - % gDa/sf] pQ/ lbg' xf]nf_ @= kof{] |
| $=s | of] k\|fo slt eO/xG5 < | ! = Pdbd sd -ut rf/ xKtfdf Ps jf b'Ok6s_  @ = slxn]sfxLF -ut rf/ xKtfdf tLg b]lv bz k6d;Dd_  # = k\|fo -ut rf/ xKtfdf bz k6s eGbf w]/}_ |
| %. | Uft rf/ xKtfdf, s] xh'/ jf xh'/sf] kl/jf/sf] ;b:onfO{ vfBfGgsf] k\|;:t ;'ljwf gePsf] eP/ cfˆgf] z/L/nfO{ rflxg] eGbf sd dfqfdf vfglkg ug{'kof{] < | != k/]g -^ gDa/sf] pQ/ lbg' xf]nf_@= kof{] |
| %.s | of] k\|fo slt eO/xG5 < | ! = Pdbd sd -ut rf/ xKtfdf Ps jf b'Ok6s_  @ = slxn]sfxLF -ut rf/ xKtfdf tLg b]lv bz k6d;Dd_  # = k\|fo -ut rf/ xKtfdf bz k6s eGbf w]/}_ |
| ^. | Uft rf/ xKtfdf, s] xh'/ jf xh'/sf] kl/jf/sf] ;b:onfO{ vfBfGgsf] k\|;:t ;'ljwf gePsf] sf/0f yf]/} dfqfdf dfq vfglkg ug{'kof{] < | != k/]g -&gDa/sf] pQ/ lbg' xf]nf_@= kof{] |
| ^.s | of] k\|fo slt eO/xG5 < | ! = Pdbd sd -ut rf/ xKtfdf Ps jf b'Ok6s_  @ = slxn]sfxLF -ut rf/ xKtfdf tLg b]lv bz k6d;Dd_  # = k\|fo -ut rf/ xKtfdf bz k6s eGbf w]/}_ |
| &. | Uft rf/ xKtfdf, s] xh'/sf] 3/df >f]tsf] sdLn] ubf{ s'g} klg k\|sf/sf] vfBfGgsf] cefj dx;'; eof] < | != ePg - * gDa/sf] pQ/ lbg' xf]nf_@= eof] |
| &.s | of] k\|fo slt eO/xG5 < | ! = Pdbd sd -ut rf/ xKtfdf Ps jf b'Ok6s_  @ = slxn]sfxLF -ut rf/ xKtfdf tLg b]lv bz k6d;Dd_  # = k\|fo -ut rf/ xKtfdf bz k6s eGbf w]/}_ |
| *. | Uft rf/ xKtfdf, s] xh'/ jf xh'/sf] kl/jf/sf] ;b:onfO{ vfBfGgsf] cefjn] ubf{ ef]s} /ftL ;'Tg' kof{] < | != k/]g -(gDa/sf] pQ/ lbg' xf]nf_@= kof{] |
| *.s | of] k\|fo slt eO/xG5 < | ! = Pdbd sd -ut rf/ xKtfdf Ps jf b'Ok6s_  @ = slxn]sfxLF -ut rf/ xKtfdf tLg b]lv bz k6d;Dd_  # = k\|fo -ut rf/ xKtfdf bz k6s eGbf w]/}_ |
| (. | Uft rf/ xKtfdf, s] xh'/ jf xh'/sf] kl/jf/sf] ;b:onfO{ vfBfGgsf] cefjn] ubf{ Ps lbg / /ft ef]s} sf6\g' kof{] < | != k/]g -( s sf] pQ/ glbg' xf]nf_@=kof{] |
| (.s | of] k\|fo slt eO/xG5 < | ! = Pdbd sd -ut rf/ xKtfdf Ps jf b'Ok6s_  @ = slxn]sfxLF -ut rf/ xKtfdf tLg b]lv bz k6d;Dd_  # = k\|fo -ut rf/ xKtfdf bz k6s eGbf w]/}_ |
