# Supplementary material for: Determinants of severe acute malnutrition among under 5 children in Satar community of Jhapa, Nepal
Source: PLoS One. 2021 Feb 3;16(2):e0245151. doi: 10.1371/journal.pone.0245151 (PMC7857586; doi:10.1371/journal.pone.0245151)
Supplement: S2 File — (DOCX) [file pone.0245151.s002.docx]

### Questionnaire: English

**Determinants of severe acute malnutrition among under 5 children in Satar community of Jhapa, Nepal**

**B.P. Koirala Institute of Health Sciences, Dharan, Nepal.**

**Number of participants included in the pretesting of this tool: 15 (10% of total sample size: 5 cases and 10 controls)**

**Code No. ………… Date: ………………**

1. **Demographic and socio-economic factors**

| **Code** | **Questions** | **Response** |
| --- | --- | --- |
|  | **Age of child (in months)** | __________ |
|  | **Gender** | □ Male □ Female □Other |
|  | **Religion** | □ Hindu □ Christian □Muslim  □ Other |
|  | **Mother’s Educational level** | □ Illiterate □ Non-formal education  □Primary □ Secondary □SLC and above |
|  | **Father’s Educational level** | □ Illiterate □ Non-formal education  □Primary □ Secondary □SLC and above |
|  | **Mother’s age at birth of the child (in yrs)** |  |
|  | **Family Income** | __________________(monthly)  __________________(yearly) |
|  | **No. of Family members** |  |
|  | **Birth Interval** | □ First Birth □ More than 2 years  □ Less than 2 years |

**B) Feeding practices:**

| **Code** | **Questions** | **Response** |
| --- | --- | --- |
|  | **Colostrum feeding** | □ Yes □ No |
|  | **Initiation of Breast Feeding** | □ Within 1 hour of birth  □ After 1 hour of birth |
|  | **Exclusive Breast Feeding upto 6 months** | □ Done □ Not Done |
|  | **Initiation of complementary feeding** | □ Before 6 months □ At 6 months  □ After 6months  □ Other |
|  | **Frequency of Breast Feeding** | □ <8/day □ >8/day |
|  | **Bottle Feeding** | □ Yes □ No |

**Anthropometric measurement**

| Height | ………….in cms |
| --- | --- |
| Weight | …………..kg |

**Household Food Insecurity Access Scale (HFIAS)**

| NO | QUESTION | RESPONSE OPTIONS |
| --- | --- | --- |
| 1. | In the past four weeks, did you worry that your household would not have enough food? | 0 = No (skip to Q2) 1=Yes |
| 1.a | How often did this happen? | 1 = Rarely (once or twice in the past four weeks)  2 = Sometimes (three to ten times in the past four weeks)  3 = Often (more than ten times in the past four weeks) |
| 2. | In the past four weeks, were you or any household member not able to eat the kinds of foods you preferred because of a lack of resources? | 0 = No (skip to Q3) 1=Yes |
| 2.a | How often did this happen? | 1 = Rarely (once or twice in the past four weeks)  2 = Sometimes (three to ten times in the past four weeks)  3 = Often (more than ten times in the past four weeks) |
| 3. | In the past four weeks, did you or any household member have to eat a limited variety of foods due to a lack of resources? | 0 = No (skip to Q4) 1 = Yes |
| 3.a | How often did this happen? | 1 = Rarely (once or twice in the past four weeks)  2 = Sometimes (three to ten times in the past four weeks)  3 = Often (more than ten times in the past four weeks) |
| 4. | In the past four weeks, did you or any household member have to eat some foods that you really did not want to eat because of a lack of resources to obtain other types of food? | 0 = No (skip to Q5) 1 = Yes |
| 4.a | How often did this happen? | 1 = Rarely (once or twice in the past four weeks)  2 = Sometimes (three to ten times in the past four weeks)  3 = Often (more than ten times in the past four weeks) |
| 5. | In the past four weeks, did you or any household member have to eat a smaller meal than you felt you needed because there was not enough food? | 0 = No (skip to Q6) 1 = Yes |
| 5.a | How often did this happen? | 1 = Rarely (once or twice in the past four weeks)  2 = Sometimes (three to ten times in the past four weeks)  3 = Often (more than ten times in the past four weeks) |
| 6. | In the past four weeks, did you or any other household member have to eat fewer meals in a day because there was not enough food? | 0 = No (skip to Q7) 1 = Yes |
| 6.a | How often did this happen? | 1 = Rarely (once or twice in the past four weeks)  2 = Sometimes (three to ten times in the past four weeks)  3 = Often (more than ten times in the past four weeks) |
| 7. | In the past four weeks, was there ever no food to eat of any kind in your household because of lack of resources to get food? | 0 = No (skip to Q8) 1 = Yes |
| 7.a | How often did this happen? | 1 = Rarely (once or twice in the past four weeks)  2 = Sometimes (three to ten times in the past four weeks)  3 = Often (more than ten times in the past four weeks) |
| 8. | In the past four weeks, did you or any household member go to sleep at night hungry because there was not enough food? | 0 = No (skip to Q9) 1 = Yes |
| 8.a | How often did this happen? | 1 = Rarely (once or twice in the past four weeks)  2 = Sometimes (three to ten times in the past four weeks)  3 = Often (more than ten times in the past four weeks) |
| 9. | In the past four weeks, did you or any household member go a whole day and night without eating anything because there was not enough food? | 0 = No (questionnaire is finished) 1 = Yes |
| 9.a | How often did this happen? | 1 = Rarely (once or twice in the past four weeks)  2 = Sometimes (three to ten times in the past four weeks)  3 = Often (more than ten times in the past four weeks) |
