# Supplementary material for: Determinants of severe acute malnutrition among under 5 children in Satar community of Jhapa, Nepal
Source: PLoS One. 2021 Feb 3;16(2):e0245151. doi: 10.1371/journal.pone.0245151 (PMC7857586; doi:10.1371/journal.pone.0245151)
Supplement: S3 File — (DOCX) [file pone.0245151.s003.docx]

## Calculation of the sample size:

The sample size was calculated, based on the study from Bara, Determinants of severe acute malnutrition among children under 5 years of age in Nepal: a community based case–control study [1].

Where the prevalence of controls who were bottle fed = Proportion of controls exposed= P_controls exp=_ 18%, (i.e. p_1_ = 18% = 0.18) and q_1_ = 1 - 0.18 = 0.82

Now, OR = 4.56

As this study considered 5% significance (α = 0.05) and 90% power to determine the sample size, We have, Z_α/2_ = 1.96 (at 5% significance level) And, for 90% power, Z_β_=1.28

For case control study, the sample size was calculated using the formula given by Schlesselman 1982 [2].

p_2_ = Proportion of case exposed= P_case exp_= $\frac{OR*Pcontrols exp}{Pcontrols exp * (OR-1)+1}$

= $\frac{4.56*0.18}{0.18 * (4.56-1)+1}$ = 0.5

q_2_ = 1-p_2_ =1- 0.5 = 0.5

Also,

**Average proportion exposed =**‾p = $\frac{p1+p2}{2}$ = $\frac{0.18+0.5}{2}$ = 0.34

‾q **=** 1-‾p = 1- 0.34 = 0.66

Then, the values in formula were put for calculation of sample size of case control study,

n = $\frac{{\{Z_{\frac{\alpha}{2}}\sqrt{\left( 2\bar{p}\bar{q} \right)} + Z_{\beta}\sqrt{(p_{1}q_{1}+p_{2}q_{2})}\}}^{2}}{{(p_{1}-p_{2})}^{2}}$

= $\frac{{\{{1.96}\sqrt{\left( 2\times0.34\times0.66 \right)} + 1.28\sqrt{(0.18\times0.82 +0.5\times0.5)}\}}^{2}}{{(0.18 -0.5)}^{2}}$ = 43

Thus, sample size required for each group was 43 children.

Since the ratio of selecting cases and controls was 1:2. So the minimum sample size required for the study was calculated to be 43 cases and 86 controls. However, after adding 10% non-response rate overall 50 cases and 100 controls samples were collected.

References:

1. Pravana NK, Piryani S, Chaurasiya SP, Kawan R, Thapa RK, Shrestha S. Determinants of severe acute malnutrition among children under 5 years of age in Nepal: a community-based case–control study. BMJ Open. 2017 Aug;7(8):e017084.
2. Schlesselman, J.J. (1982) Case-control studies, design, conduct, analysis. Oxford University Press, New York.
